# The Role of Human Endogenous Retroviruses in Cancer Immunotherapy of the Post-COVID-19 World

**DOI:** 10.3390/cancers15225321

**Published:** 2023-11-07

**Authors:** Stella Logotheti, Thorsten Stiewe, Alexandros G. Georgakilas

**Affiliations:** 1 DNA Damage Laboratory, Physics Department, School of Applied Mathematical and Physical Sciences, National Technical University of Athens (NTUA), Zografou, 15780 Athens, Greece; 2 Institute of Molecular Oncology, Philipps-University, 35043 Marburg, Germany; stiewe@uni-marburg.de; 3 German Center for Lung Research (DZL), Universities of Giessen and Marburg Lung Center (UGMLC), 35043 Marburg, Germany; 4 Genomics Core Facility, Philipps-University, 35043 Marburg, Germany; 5 Institute for Lung Health (ILH), Justus Liebig University, 35392 Giessen, Germany

At the outbreak of the COVID-19 global crisis, diverse scientific groups suggested that this unprecedented emergency could act as a ‘blessing in disguise’. In the aftermath of the pandemic, cancer immunotherapy is unexpectedly ranked among the particularly benefited fields. Lessons learned from the real-life safety and efficacy profiles of the vaccines developed against SARS-CoV-2 are currently being applied in the field of cancer. At the same time, the introduction of relevant vaccine technology in clinical cancer trials has pinpointed a number of novel challenges, including the selection of appropriate targets, the prediction of responses, and the improvement of tolerance and specificity. In this Editorial, we draw attention to important recent articles that describe these tectonic changes in cancer immunotherapeutics and indicate the perspectives for human endogenous retroviruses (HERV) regarding further improving personalized patient management.

Immunotherapy has induced a rapid paradigm shift in clinical oncology. A key reason for the success of immunological drugs is that, instead of directly attacking tumors as cytotoxic drugs do, they ally with a person’s own immune system and train it to recognize and destroy cancer cells, thus establishing durable responses. A well-known blockbuster, linked with the Nobel Prize in Medicine in 2018, is the FDA-approved immune checkpoint inhibitors (ICIs), which release the brakes that prevent T lymphocytes from killing cancer cells. Intriguingly, the pandemic propelled mRNA vaccines, a distinct class of immunotherapeutics, into the limelight as a cost-effective means to harness our immune systems against this global viral threat [1]. Encouraged on the one hand by the favorable data on the safety and efficacy of mRNA vaccines for SARS-CoV-2, and on the other hand by the success story of ICIs, researchers are now using the ‘pandemic heritage’ as a springboard to design personalized, mRNA vaccine-based treatments for several cancer types [1]. Advantageously, mRNA molecules induce both humoral and cellular immune responses, and are well-tolerated and easily degradable, while their production is fast, scalable, and inexpensive. The results from early clinical trials with mRNA vaccines as monotherapy and in combination with ICIs are auspicious [2]. A growing number of recent studies highlight the evolution and progress of cancer mRNA vaccines in the clinical setting [1,3,4], as well as the development of delivery systems, like lipid or polymeric nanoparticles, and protamines [5]. A crucial aspect for the design of mRNA vaccines is the identification of targets, which ideally need to be immunogenic and exhibit tumor-restricted expression [5].

Nevertheless, responses to immunotherapeutics vary widely, since some patients achieve superior outcomes, while others do not really benefit. Long-term updates show a sustained clinical benefit for melanoma patients treated with ICIs such as nivolumab or pembrolizumab or a combination of nivolumab and ipilimumab. At the polar opposite, about 40–50% of patients acquire resistance within five years from the onset of ICI therapy [6]. On a similar note, Huang and colleagues reported a 54.6% primary resistance rate in 108 patients with advanced non-small cell lung cancer (NSCLC), and found that female gender, a neutrophil-lymphocyte ratio > 3, and administration of ICI beyond first-line treatment are significant predictors of ICI unresponsiveness [7]. Apart from the expression of checkpoint molecules like PD-1 and PD-L1, the prediction of responsiveness is often guided by tumor mutational burden (TMB). Higher TMB produces more neoantigens, thereby improving T-cell recognition and ICI outcomes. Solid tumors bearing a TMB with ≥10 mutations per megabase received agnostic approval for pembrolizumab [8]. However, inconsistencies between TMB and ICI patient response across all cancer types redefined a clinical need for complementary predictive markers [8,9,10,11]. Recent re-evaluation of the IMpower133 study, a pivotal trial demonstrating the survival advantage of combining the checkpoint inhibitor atezolizumab with carboplatin and etoposide in first-line extensive-stage small cell lung cancer patients, underscored that both blood-based TMB and PD-L1 status should not be relied upon for patient treatment decisions, as current evidence suggests neither biomarker is consistently predictive [12]. Hence, post-pandemic advances in the burgeoning field of cancer immunotherapy go hand-in-hand with a need for predicting and/or improving clinical benefits on a personalized patient basis. At the dawn of the new era of mRNA cancer vaccines, the times call imperatively for identifying reliable biomarkers for patient stratification, as well as novel targets that boost treatment efficiency. In this changing landscape, human endogenous retroviruses (HERVs) are entering the stage to aid cancer immunotherapy to deliver on its promise.

HERVs constitute 8% of the human genome and have attracted increasing attention due to their association with cancer development and immunotherapy. They are remnants of ancient retroviruses, which entered the germline by infecting our distant ancestors. In the largest in silico survey on full-length HERVs across 49 primates, Li Y et al. traced their origin back to nonhuman primates and reported that some HERVs originated before the speciation of Hominidae, Hylobates, and Old-World monkeys [13]. Over time, HERVs lost their potential for intercellular mobility, became non-infectious, co-evolved with the human genome, and acquired essential physiological functions. By rewiring with the host transcriptional networks, HERVs undertook new roles as promoters and enhancers of indispensable genes during early embryonic development. During oncogenesis, their promoter/enhancer activity is ‘hijacked’ to drive the expression of oncogenes, a process termed onco-exaptation. Furthermore, HERVs remain silent in normal tissues, mainly through epigenetic mechanisms, but their expression becomes de-suppressed in tumors [14]. A study published in *Cancers* in July 2023 comprehensively analyzed the expression of several classes of HERVs in hepatocellular carcinomas (HCC). By applying RNA sequencing on 254 HCC tissues and their 34 matched normal tissue controls, the authors described 3357 locus-specific activations of HERVs, 180 of which were correlated with poor patient survival [15]. Similarly, HERV-K, the most recently integrated retrovirus in the human genome, is typically transcriptionally silenced in normal adult cells, but becomes re-activated in melanoma, teratocarcinoma, osteosarcoma, breast cancer, lymphoma, and ovary and prostate cancers and promotes cancer stemness and dedifferentiation [16].

Strikingly, HERVs have developed a multifaceted crosstalk with the immune system of their human hosts [14,17]. During their co-evolution, HERVs shaped the host immune system, which, in turn, controls their expression [17]. Despite the fact that HERV-mediated gene dysregulation promotes oncogenesis through exaptation, the expression of HERV products renders tumors visible to the immune system. In particular, proviral HERV DNA is transcribed to either double-stranded RNA (dsRNA) that is sensed as a “danger signal” by pattern recognition receptors or to mRNA that is translated into proteins [14]. On the one hand, the dsRNA signals elicit host innate immunity via the induction of viral defense, a phenomenon known as viral mimicry. On the other hand, the produced proteins act as tumor-associated antigens and sensitize tumors to T cell-mediated immunological recognition [14]. In hematological cancer patients, CD8+ T cell populations were shown to recognize 29 HERV-derived peptides from 18 different HERV loci, among which HERVH-5, HERVW-1, and HERVE-3 have more profound responses. These T cells were found in 17 of the 34 patients but were infrequent in healthy donors [18]. Moreover, machine-learning approaches identified shared T-cell epitopes that derive from HERV antigens which are specifically overexpressed in different breast cancer subtypes and prime antitumor cytotoxic T-cell clones of high avidity [19]. Overall, the immunogenic potency of HERVs in combination with their tumor-specific profiles have important implications for cancer immunotherapy.

Several groups have, independently, underlined that tumor-specific reactivation of HERVs generates signatures that predict responses to immunotherapy. Clinical studies have associated the expression of HERVs and response to ICIs not only in NSCLC patients [20], who exhibit typically high TMB, but also in renal cell carcinoma patients, who have a relatively low TMB [21]. Consistently, analysis of genomic, transcriptomic, and clinical data across an integrated cohort of 199 patients with metastatic breast, colorectal, and pancreatic ductal adenocarcinoma tumors showed that within each cancer type, there is a subgroup of tumors with viral mimicry phenotypes in which increased HERV levels are indicative for immunogenicity [22]. The recurrent correlations of HERVs with T cell-mediated cytotoxicity can be explained, at least in part, if we consider that besides tumor-specific neoantigens, the immune system also recognizes tumor-associated antigens, that derive from ‘off-context’ gene re-activations rather than nucleotide mutations. These include, but are not limited to, differentiation antigens, cancer-testis antigens and reactivated HERVs. As a representative example, human and murine lung adenocarcinomas present tertiary lymphoid structures which exhibit correlations with ICI response and lead to the generation of antibodies primarily targeting endogenous retroviruses aberrantly expressed by tumor cells [23]. Consequently, tumors enriched in these alternative sources of tumor-specific antigens may be detected by immune cells, even if they have a low TMB [24]. Furthermore, emerging notions assert that immunoreactivity depends on the quality of neoantigens, rather than the quantity, since cancer patients with better survival and stronger T-cell activity bear tumors with fewer, but more immunogenic, neoantigens [25]. In an analogous manner, the HERV-derived products might represent a reservoir of ‘high-quality’ antigenic molecules that potentially influence tumor immune responses irrespective of the presence of neoantigens or TMB. In this regard, their consistent immunogenic profiles might hold potential to be used as indicators of ICI response, to surrogate or supplement existing clinical predictors.

It is noteworthy that HERVs’ re-activation not only predicts but also increases responsiveness to ICI therapy. For example, a vaccine encoding for a region of the envelope (Env) protein of HERV (described as an immunosuppressive domain, ISD) has been developed and tested in the preclinical setting. Mutated ISD enhanced T-cell immunogenicity in both prime and prime-boost vaccinations and, when combined with ICI therapy, it eradicated colorectal tumors in mice [26]. Additionally, the group of Selivanova provided robust in vivo evidence that HERV-mediated viral mimicry is triggered in tumors by pharmacological activators of p53 and eventually overcomes resistance to ICIs. As p53 is the most frequently mutated gene across cancer types and a ‘holy grail’ of anticancer targeting, this seminal finding can be a game-changer in the design of p53-based therapies [27].

In conclusion, HERV products could be invaluable as predictors of ICI response and/or targets for breakthrough immunotherapeutic formulations. Intriguingly, therapeutic responses increase when ICIs are combined with mRNA vaccines. In KEYNOTE-942 (NCT03897881), an ongoing open-label phase IIb trial on completely resected stage III/IV melanoma, patients who received a combination of a mRNA vaccine encoding 20 different mutated neoantigens (mRNA-4157/V940) plus ICI (pembrolizumab) showed 44% lower risk of disease relapse or death versus those who received pembrolizumab monotherapy [3]. Subsequently, mRNA vaccines against HERVs could be considered as adjuvant agents in combination regimens with ICIs. To minimize putative off-target toxicity on tissues that may normally express HERV antigens, such as human placenta, stem cells and immune cells [28], the mRNA vaccine technology can stand on the shoulders of recent advances in computational pipelines. These pipelines entail, for example, the construction of single-cell transcriptomic atlases of cancer patients and tissue from healthy individuals that guide the prediction of safe target antigens that are expressed on malignant cells but lacking on healthy cells [29]. The journey of HERVs in the field of immunotherapy has just begun, and both opportunities and challenges are ahead. Future fruitful research will help to untangle the interplay of HERVs with the immune system and unleash their translational potential for precision oncology.

## References

[1] Krause W. (2023). mRNA-From COVID-19 Treatment to Cancer Immunotherapy. Biomedicines.

[2] Lorentzen C.L., Haanen J.B., Met Ö., Svane I.M. (2022). Clinical advances and ongoing trials on mRNA vaccines for cancer treatment. Lancet Oncol..

[3] Bafaloukos D., Gazouli I., Koutserimpas C., Samonis G. (2023). Evolution and Progress of mRNA Vaccines in the Treatment of Melanoma: Future Prospects. Vaccines.

[4] Bidram M., Zhao Y., Shebardina N.G., Baldin A.V., Bazhin A.V., Ganjalikhany M.R., Zamyatnin A.A., Ganjalikhani-Hakemi M. (2021). mRNA-Based Cancer Vaccines: A Therapeutic Strategy for the Treatment of Melanoma Patients. Vaccines.

[5] Sun H., Zhang Y., Wang G., Yang W., Xu Y. (2023). mRNA-Based Therapeutics in Cancer Treatment. Pharmaceutics.

[6] Hassel J.C., Zimmer L., Sickmann T., Eigentler T.K., Meier F., Mohr P., Pukrop T., Roesch A., Vordermark D., Wendl C. (2023). Medical Needs and Therapeutic Options for Melanoma Patients Resistant to Anti-PD-1-Directed Immune Checkpoint Inhibition. Cancers.

[7] Huang Y., Zhao J.J., Soon Y.Y., Kee A., Tay S.H., Aminkeng F., Ang Y., Wong A.S.C., Bharwani L.D., Goh B.C. (2023). Factors Predictive of Primary Resistance to Immune Checkpoint Inhibitors in Patients with Advanced Non-Small Cell Lung Cancer. Cancers.

[8] Xavier C.B., Lopes C.D.H., Awni B.M., Campos E.F., Alves J.P.B., Camargo A.A., Guardia G.D.A., Galante P.A.F., Jardim D.L. (2022). Interplay between Tumor Mutational Burden and Mutational Profile and Its Effect on Overall Survival: A Pilot Study of Metastatic Patients Treated with Immune Checkpoint Inhibitors. Cancers.

[9] Jardim D.L., Goodman A., de Melo Gagliato D., Kurzrock R. (2021). The Challenges of Tumor Mutational Burden as an Immunotherapy Biomarker. Cancer Cell.

[10] McGrail D.J., Pilié P.G., Rashid N.U., Voorwerk L., Slagter M., Kok M., Jonasch E., Khasraw M., Heimberger A.B., Lim B. (2021). High tumor mutation burden fails to predict immune checkpoint blockade response across all cancer types. Ann. Oncol..

[11] Lawlor R.T., Mattiolo P., Mafficini A., Hong S.M., Piredda M.L., Taormina S.V., Malleo G., Marchegiani G., Pea A., Salvia R. (2021). Tumor Mutational Burden as a Potential Biomarker for Immunotherapy in Pancreatic Cancer: Systematic Review and Still-Open Questions. Cancers.

[12] Liu S.V., Reck M., Mansfield A.S., Mok T., Scherpereel A., Reinmuth N., Garassino M.C., De Castro Carpeno J., Califano R., Nishio M. (2021). Updated Overall Survival and PD-L1 Subgroup Analysis of Patients With Extensive-Stage Small-Cell Lung Cancer Treated With Atezolizumab, Carboplatin, and Etoposide (IMpower133). J. Clin. Oncol..

[13] Li Y., Zhang G., Cui J. (2022). Origin and Deep Evolution of Human Endogenous Retroviruses in Pan-Primates. Viruses.

[14] Petrizzo A., Ragone C., Cavalluzzo B., Mauriello A., Manolio C., Tagliamonte M., Buonaguro L. (2021). Human Endogenous Retrovirus Reactivation: Implications for Cancer Immunotherapy. Cancers.

[15] Chang Y.S., Hsu M.H., Chung C.C., Chen H.D., Tu S.J., Lee Y.T., Yen J.C., Liu T.C., Chang J.G. (2023). Comprehensive Analysis and Drug Modulation of Human Endogenous Retrovirus in Hepatocellular Carcinomas. Cancers.

[16] Rivas S.R., Valdez M.J.M., Govindarajan V., Seetharam D., Doucet-O’Hare T.T., Heiss J.D., Shah A.H. (2022). The Role of HERV-K in Cancer Stemness. Viruses.

[17] Alcazer V., Bonaventura P., Depil S. (2020). Human Endogenous Retroviruses (HERVs): Shaping the Innate Immune Response in Cancers. Cancers.

[18] Saini S.K., Ørskov A.D., Bjerregaard A.M., Unnikrishnan A., Holmberg-Thydén S., Borch A., Jensen K.V., Anande G., Bentzen A.K., Marquard A.M. (2020). Human endogenous retroviruses form a reservoir of T cell targets in hematological cancers. Nat. Commun..

[19] Bonaventura P., Alcazer V., Mutez V., Tonon L., Martin J., Chuvin N., Michel E., Boulos R.E., Estornes Y., Valladeau-Guilemond J. (2022). Identification of shared tumor epitopes from endogenous retroviruses inducing high-avidity cytotoxic T cells for cancer immunotherapy. Sci. Adv..

[20] Lecuelle J., Favier L., Fraisse C., Lagrange A., Kaderbhai C., Boidot R., Chevrier S., Joubert P., Routy B., Truntzer C. (2022). MER4 endogenous retrovirus correlated with better efficacy of anti-PD1/PD-L1 therapy in non-small cell lung cancer. J. Immunother. Cancer.

[21] Panda A., de Cubas A.A., Stein M., Riedlinger G., Kra J., Mayer T., Smith C.C., Vincent B.G., Serody J.S., Beckermann K.E. (2018). Endogenous retrovirus expression is associated with response to immune checkpoint blockade in clear cell renal cell carcinoma. JCI Insight.

[22] Topham J.T., Titmuss E., Pleasance E.D., Williamson L.M., Karasinska J.M., Culibrk L., Lee M.K.C., Mendis S., Denroche R.E., Jang G.H. (2020). Endogenous Retrovirus Transcript Levels Are Associated with Immunogenic Signatures in Multiple Metastatic Cancer Types. Mol. Cancer Ther..

[23] Ng K.W., Boumelha J., Enfield K.S.S., Almagro J., Cha H., Pich O., Karasaki T., Moore D.A., Salgado R., Sivakumar M. (2023). Antibodies against endogenous retroviruses promote lung cancer immunotherapy. Nature.

[24] Wolf M.M., Rathmell W.K., de Cubas A.A. (2023). Immunogenicity in renal cell carcinoma: Shifting focus to alternative sources of tumour-specific antigens. Nat. Rev. Nephrol..

[25] Łuksza M., Sethna Z.M., Rojas L.A., Lihm J., Bravi B., Elhanati Y., Soares K., Amisaki M., Dobrin A., Hoyos D. (2022). Neoantigen quality predicts immunoediting in survivors of pancreatic cancer. Nature.

[26] Daradoumis J., Ragonnaud E., Skandorff I., Nielsen K.N., Bermejo A.V., Andersson A.M., Schroedel S., Thirion C., Neukirch L., Holst P.J. (2023). An Endogenous Retrovirus Vaccine Encoding an Envelope with a Mutated Immunosuppressive Domain in Combination with Anti-PD1 Treatment Eradicates Established Tumours in Mice. Viruses.

[27] Zhou X., Singh M., Sanz Santos G., Guerlavais V., Carvajal L.A., Aivado M., Zhan Y., Oliveira M.M.S., Westerberg L.S., Annis D.A. (2021). Pharmacologic Activation of p53 Triggers Viral Mimicry Response Thereby Abolishing Tumor Immune Evasion and Promoting Antitumor Immunity. Cancer Discov..

[28] Denner J. (2021). Endogenous retroviruses expressed in human tumours cannot be used as targets for anti-tumour vaccines. Transl. Oncol..

[29] Gottschlich A., Thomas M., Grünmeier R., Lesch S., Rohrbacher L., Igl V., Briukhovetska D., Benmebarek M.R., Vick B., Dede S. (2023). Single-cell transcriptomic atlas-guided development of CAR-T cells for the treatment of acute myeloid leukemia. Nat. Biotechnol..

